# Association of *ADIPOQ* gene with type 2 diabetes and related phenotypes in African American men and women: the Jackson Heart Study

**DOI:** 10.1186/s12863-015-0319-4

**Published:** 2015-12-23

**Authors:** Sharon K. Davis, Ruihua Xu, Samson Y. Gebreab, Pia Riestra, Amadou Gaye, Rumana J. Khan, James G. Wilson, Aurelian Bidulescu

**Affiliations:** National Human Genome Research Institute, Genomics of Metabolic, Cardiovascular and Inflammatory Disease Branch, Social Epidemiology Research Unit, 10 Center Drive, Bethesda, MD 20892 USA; Department of Physiology, University of Mississippi Center, 2500 N State St, Jackson, MS 39216 USA; Indiana University Bloomington, School of Public Health, 1025 E. 7th St, Suite 111, Bloomington, IN 47405 USA

**Keywords:** Adiponectin, Type 2 diabetes, ADIPOQ gene, African Americans

## Abstract

**Background:**

African Americans experience disproportionately higher prevalence of type 2 diabetes and related risk factors. Little research has been done on the association of *ADIPOQ* gene on type 2 diabetes, plasma adiponectin, blood glucose, HOMA-IR and body mass index (BMI) in African Americans. The objective of our research was to assess such associations with selected SNPs. The study included a sample of 3,020 men and women from the Jackson Heart Study who had *ADIPOQ* genotyping information. Unadjusted and adjusted regression models with covariates were used with type 2 diabetes and related phenotypes as the outcome stratified by sex.

**Results:**

There was no association between selected *ADIPOQ* SNPs with type 2 diabetes, blood glucose, or BMI in men or women. There was a significant association between variant rs16861205 and lower adiponectin in women with minor allele A in the fully adjusted model (β(SE) *p* = −.13(0.05), 0.003). There was also a significant association with variant rs7627128 and lower HOMA-IR among men with minor allele A in the fully adjusted model (β(SE) *p* = −0.74(0.20), 0.0002).

**Conclusions:**

These findings represent new insights regarding the association of *ADIPOQ* gene and type 2 diabetes and related phenotypes in African American men and women.

## Background

Type 2 diabetes is more prevalent among African Americans when compared to most racial/ethnic groups in the US–even after taking into account socioeconomic status (SES), prevalence and severity of hypertension and access to health care [1–4]. African Americans also have a higher prevalence of elevated A1C hemoglobin, fasting blood glucose, insulin resistance and obesity which are risk factors for type 2 diabetes [1, 5, 6]. Adverse behavioral lifestyle, such as poor diet and physical inactivity, are contributing factors associated with type 2 diabetes. African Americans have an overall worse lifestyle profile and lower SES [1, 7].

Plasma adiponectin levels are inversely correlated with type 2 diabetes, blood glucose, insulin resistance and obesity [8]. Adiponectin is an adipose tissue-specific hormone that is responsible for increasing energy expenditure and lipid catabolism as well as enhancing fatty acid oxidation and insulin sensitivity [9]. African Americans present with lower levels of adiponectin and have more severe type 2 diabetes phenotypes [10]. The adiponectin gene (*ADIPOQ*) located at position 3q27 has been established as the main genetic determinant of plasma adiponectin levels with an inheritance genetic component between 30 to 70 % [11]. The *ADIPOQ* gene spans 1.579 kb and contains 3 exons. The translation start point is located in exon 2 [12]. Several single nucleotide polymorphisms (SNPs) located in *ADIPOQ* have been associated with adiponectin serum levels, body adiposity and metabolic alterations making this gene a candidate for type 2 diabetes and associated traits [12–14]. A limited number of studies have investigated the association of genetic variants in the adiponectin gene with type 2 diabetes and its related phenotypes in African Americans [15–19]. Many of these studies have yielded conflicting results due to small sample size, inclusion of only one gender, and the confounding effect of unadjusted population structure and behavioral lifestyle factors. The objective of the current study was to assess the association of SNPs in *ADIPOQ* with type 2 diabetes, level of plasma adiponectin, blood glucose, insulin resistance and body mass index (BMI) in African American men and women with adjustments for biological, behavioral and socioeconomic factors. We hypothesized that, after adjustments, the variants related with adiponectin would be associated with type 2 diabetes and its related phenotypes.

## Research design and Methods

### Study subjects

Cross-sectional data from the Jackson Heart Study (JHS) was used in this study. The JHS is a single-site, community-based study of risk factors and causes of heart disease in adult African Americans. A total of 5,301 non-institutionalized African Americans aged 21–95 years residing in three contiguous counties surrounding Jackson, MS were recruited, interviewed and examined by certified technicians according to standardized protocols at baseline from 2000–2004 [20, 21]. All of the participants gave written informed consent to participate. The clinic visits included the collection of data on sociodemographics, anthropometry, survey of medical history, cardiovascular behavioral risk factors and blood and urine for biological risk factors. The data for this study includes a total of 3,020 men and women with complete DNA and total plasma adiponectin conducted on serum specimens collected at baseline from 2000–2004. These 3,020 participants gave consent for genetic analyses and were genotyped separately in the CARe consortium in 2006 using Affymetrix 6.0 platform [22]. This study was approved by the Institutional Review Board of the National Institutes of Health and the study protocol was approved by the Institutional Review Boards of the participating JHS institutions, including the University of Mississippi Medical Center, Jackson State University and Tougaloo College.

### Outcome phenotypes

The main outcomes of the study were type 2 diabetes, plasma adiponectin, blood glucose, homeostatis model assessment–insulin resistance (HOMA-IR), and BMI. Type 2 diabetes was defined as fasting plasma glucose ≥ 126 mg/dL or self-reported use of insulin or oral hypoglycemic medications [23]. Adiponectin measurement was derived from venous blood samples drawn from each participant after more than 8 h of fasting. Vials of serum were stored at the JHS central repository in Minneapolis, MN at −80 °C until assayed. Adiponectin concentration was measure as total plasma adiponectin by ELISA system (R & D Systems; Minneapolis, MN). The inter-assay coefficient of variation was 8.8 %. No biological degrading has been described using stored specimens, indicating a high validity for measurement [24]. Fasting plasma glucose and fasting insulin were measured using standard laboratory techniques. The HOMA-IR was calculated as [insulin (microunits per milliliter) x fasting blood glucose (millimoles per liter)]/22.5. Insulin resistance was defined as a HOMA-IR in the highest quartile of its distribution [25]. Body mass index was based on standing height and weight measured on a balance scale in lightweight clothing without shoes or constricting garments with weight recorded to the nearest 0.5 kg and calculated as weight in kilograms by height in meters squared (kg/m^2^).

### Primary predictor: SNP selection genotyping and imputation

A candidate gene approach for the selection of the genetic variants was used. The tagging approach was applied to the entire set of common genetic variants in the *ADIPOQ* gene (5kb upstream of the first exon and 5kb downstream of the last exon of the gene) with minor allele frequency (MAF) ≥1 % in Yoruba population (YRI) from the International HapMap Project [26]. SNPs were chosen based on their ability to capture genetic information for the YRI population. Tagging SNPs were selected by the Tagger algorithm available through Haploview using a pairwise SNP selection and captured an inter-SNP r^2^ value of > 0.80 for known polymorphisms in the region. This process resulted in a selection of 15 tagging SNPs for *ADIPOQ* with a mean r^2^ of 0.969 of the selected SNPs. This selection captures a high degree (over 95 %) of the known variability in this gene. IMPUTE2 software and reference phased data from the 1000G project were used for genotype imputation to infer *ADIPOQ* SNPs genotypes [27, 28]. SNP-level quality control metrics were applied prior to downstream analyses and included the following: call rate ≥ 95 %, MAF ≥1 %, Hardy-Weinberg equilibrium (HWE) Bonferroni correction = *p* ≥ 0.003, and quality measures for imputed SNPs of r^2^ ≥ 0.3. Of the 15 SNPS, 3 were excluded because they were not available in the JHS data, and an additional 4 were excluded because they that did not meet the HWE criteria-resulting in eight SNPs for subsequent analyses.

### Covariates

Information on key covariates, which are known risk factors for type 2 diabetes and related phenotypes, was obtained from baseline examination. Age was derived from self-reported date-of-birth. Proportion of European Ancestry (PEA) for each participant was calculated using HAPMIX supported by the CARe consortium [22, 29–31]. The proportion of global European ancestry estimates for the study has a median of 16.0 % and interquartile range of 15 %.

Biological risk factor measures included low-density lipoprotein (LDL), high-density lipoprotein (HDL), triglyceride, C-reactive protein (CRP), plasma leptin, blood glucose, and HOMA-IR. Behavioral risk factors included smoking status, physical activity, BMI, and alcohol consumption. Fasting LDL, HDL, triglyceride and blood glucose were assessed using standard laboratory techniques. Fasting CRP was measured using immunturbidimetric CRP-Latex assay from Kamiya Biomedical Company following manufacturer’s high-sensitivity protocol [32]. The inter-assay coefficients of variation on control samples repeated in each assay were 4.5 and 4.4 % at CRP concentration of 0.45 and 1.56 mg/dL, respectively. The reliability coefficient for masked quality-control replicates was 0.95 for the CRP assay. Fasting leptin was collected via venous blood samples drawn from each participant and analyzed with Human Leptin PIA kit (LINCO Research, St Charles, MI, USA) [33]. Acceptable coefficient of variation was 10 % [33]. Insulin resistance status was estimated with the HOMA as previously described [25]. Smoking status was defined as current smoker and non-smoker. Physical activity was assessed with a physical activity survey instrument comprised of 4 domains (active living, work, home and garden, sport and exercise). A total score was the sum of these domains with a maximum of 24. A higher score indicates a higher level of total physical activity. The calculation of BMI was previously described. Alcohol consumption status was defined as “yes” if participant reported ever consuming alcohol and “no” for those reporting never consuming alcohol. Socioeconomic status (SES) was based on self-reported level of educational attainment - < high school, high school or graduate education equivalency diploma GED), some college or vocational school, bachelors or associate degree, post-college experience.

### Statistical analysis

All analyses were stratified by sex because of the differential prevalence of phenotypes. Baseline characteristics of the study sample were conducted by sex using *t*-test for continuous variables and chi-square for categorical variables. Hardy-Weinberg equilibrium tests for each of the *ADIPOQ* SNPs were analyzed using chi-square test. We then used logistic regression to assess the association between type 2 diabetes and each *ADIPOQ* SNP and linear regression was used to examine the associations of each *ADIPOQ* SNP with plasma, adiponectin, blood glucose, HOMA-IR, and BMI. Six sequential cumulative models, stratified by sex, were fitted for each phenotype with minor allele as the reference. Model 1 included each SNP as the primary predictor (unadjusted), model 2 included age, model 3 included PEA, model 4 included biological risk factors (LDL cholesterol, HDL cholesterol, triglyceride, CRP, plasma leptin), model 5 included behavioral risk factors (smoking status, physical activity, BMI, alcohol consumption), and model 6 included a fully adjusted model with SES based on level of educational attainment. Age, PEA, LDL cholesterol, HDL cholesterol, triglyceride, CRP, plasma leptin, blood glucose, BMI, physical activity and HOMA-IR were entered as continuous variables. Smoking status, alcohol consumption status, and SES were entered as categorical variables. Adiponectin, blood glucose, HOMA-IR and BMI were log transformed to obtain better approximations of the normal distribution prior to analysis. Multiple comparisons were controlled using Bonferroni correction which was defined a priori by dividing the significance level α = 0.05 by the number of selected *ADIPOQ* SNPS (0.05/8 = 0.00625) [34]. Therefore, a *p*-value threshold of 0.006 was used to determine statistical significance. Power analyses for the tests of association were computed using the minor allele frequencies and mean values of serum, adiponectin levels from the JHS and the effect sizes originally reported [34]. Assuming a *p* value of 0.001 and a power of 80 %, we will require 845 subjects per outcome in order to detect a 2 % of variation in adiponectin levels. Analyses were conducted using SAS version 9.3 [35]. Haplotypes were analyzed to identify haplotype blocks using linear regression in PLINK. Haplotypes with an estimated frequency <5 % were excluded from the analysis. Global *p*-values were obtained by omnibus tests jointly estimating all haplotype effects. Linear and logistic regression analysis was used for the individual haplotype association.

## Results

The sex-stratified baseline characteristics of the study population are presented in Table 1. Approximately 38 % of the sample was comprised of men and 62 % women. Women were significantly older and had a lower proportion of European ancestry (*p* <0.02 and 0.005, respectively). They also had differential levels of education compared to men (*p* <0.04). Behavioral risk factors were distributed differently between men and women. A higher proportion of men were current smokers, consumed alcohol and were more physically active (*p* <0.0001 for all). Women had a higher mean BMI (*p* <0.0001). A differential pattern was also observed regarding biological risk factors. Systolic blood pressure, DBL, LDL cholesterol, and triglyceride were higher among men (*p* < 0.03, 0.0001, 0.03, 0.0001, 0.0001, respectively). Women had higher HDL cholesterol, plasma adiponectin, leptin, CRP, and HOMA-IR (*p* < 0.0001, 0.0001, 0.0001, 0.0001, 0.0004, respectively). Additionally, a higher proportion of women had type 2 diabetes and hypertension (*p* < 0.01 and 0.009, respectively).

**Table 1:** Characteristics of men and women in the Jackson Heart Study, *N* = 3020

	Men 38.15 % (*N* = 1,152)	Women 61.85 % (*N* = 1,868)	*P*-value^*^
Demographic Factors (N)			
Age (mean ± std.)	53.96 ± 13.01	55.05 ± 12.73	0.02
PEA^┼^ (mean ± std.)	0.18 ± 0.09	0.17 ± 0.08 (1283)	0.005
Education, %	38.13 %	61.87 %	
<High school	16.74 %	16.60 %	
High school + GED^±^	19.18 %	21.49 %	
Some college + vocational school	23.98 %	22.89 %	
Bachelors + associate degree	25.02 %	21.28 %	
Post-college	15.08 %	17.73 %	0.04
Behavioral Factors (N)			
Smoking Status, % Yes	18.05 %	10.99 %	<0.0001
BMI^§^ kg/m^2^ (Mean ± std.)	30.01 ± 6.31	33.26 ± 7.81	<0.0001
Physical Activity Score (Mean ± std.)	8.55 ± 2.55	8.15 ± 2.58	<0.0001
Alcohol Consumption, % (N) Yes	59.20 %	40.30 %	<0.0001
Biological Factors (N)			
Systolic blood pressure, mmHg	127.8 ± 17.76	126.3 ± 18.34	0.03
Diastolic blood pressure, mmHg	81.53 ± 10.59	77.46 ± 10.30	<.0001
Total Cholesterol, mg/dl (Mean ± std.)	196.6 ± 40.89	199.4 ± 40.36	0.07
LDL^║^ Cholesterol, mg/dl (Mean ± std.)	127.9 ± 37.15	124.8 ± 37.12	0.03
HDL^¶^ Cholesterol, mg/dl (Mean ± std.)	45.69 ± 12.79	54.66 ± 14.71	<0.0001
Fasting Triglyceride Level, mg/dl, (Mean ± std.)	117.7 ± 100.3	101.3 ± 64.46	<0.0001
Plasma Adiponectin, ug/mL (Mean ± std.)	4.08 ± 3.38	6.04 ± 4.46	<0.0001
Plasma Leptin, ng/mL (Mean ± std.)	11.81 ± 11.12	38.53 ± 24.80	<0.0001
Blood Glucose mg/dL (Mean ± std.)	100.9 ± 33.83	100.5 ± 33.80	0.76
CRP^#^, mg/dL (Mean ± std.)	0.37 ± 1.13	0.63 ± 0.89	<.0001
HOMA-IR** (Insulin resistance) (Mean ± std.)	3.49 ± 2.17	3.85 ± 2.56	0.0004
Type II diabetes, %	17.12 %	20.87 %	0.01
Hypertension, %	65.55 %	60.84 %	0.009

^*^Two-sample *t*-test for continuous variables and chi-square for categorical variables; significance established as

*P* ≤ 0.05; *std* standard deviation

^┼^ *PEA* Percent European ancestry

^±^ *GED* Graduate equivalency diploma

^╪^ *BMI* Body mass index

^§^ *LDL* Low density lipoprotein

^║^ *HDL* High density lipoprotein

^¶^ *CRP* C-reactive protein

^**^ *HOMA-IR* Homeostasis model assessment – insulin resistance

Table 2 shows the characteristics, minor allele frequencies and HWE *p*-values for the selected *ADIPOQ* SNPs. Minor allele frequencies ranged from 6 to 43 %. All of the SNPs included in the subsequent analysis conformed to HWE.

**Table 2:** Characteristics of selected *ADIPOQ* SNPs in the adiponectin gene

ADIPOQ SNP	Location on chromosome 3^*^	Gene region	Tagging population	r^2┼^	Major/Minor allele	MAF^╪^	HWE *P*-value^§^
rs16861205	186561634	Intron 1	YRI^║^	0.99357	G/A	0.21245	0.0312
rs12495941	186568180	Intron 1	YRI	0.61244	G/T	0.35333	0.3807
rs7627128	186568799	Intron 1	YRI	0.68362	C/A	0.15217	0.7607
rs9877202	186569607	Intron 1	YRI	0.71281	A/G	0.13273	0.697
rs2036373	186570191	Intron 1	YRI	0.98257	T/G	0.06391	0.9634
rs1501299	186571123	Intron 2	YRI	0.98519	G/T	0.35484	0.5123
rs3821799	186571486	Intron 2	YRI	0.99954	T/C	0.43207	0.1744
rs9842733	186575482	3′-UTR	YRI	0.92664	A/T	0.10153	0.5974

^*^position based on NCBI Build 36

^┼^r2 refers to the measurement of SNPs imputation quality

^╪^ *MAF* Major allele frequency

^§^ *HWE* Hardy-Weinberg equilibrium; *P*-value calculated based on chi-square

^║^ *YRI* Yoruba in Ibadan, Nigeria from HAPMAP

### Association between *ADIPOQ* SNPs and phenotypes

Results are presented in Table 3. No *ADIPOQ* variant was found to be associated with type 2 diabetes in men or women in the crude or adjusted models. Results in Table 4 show no association between any of the variants and plasma adiponectin among men. However, two variants were significantly associated in women. *ADIPOQ* SNP rs16861205 was significantly associated with adiponectin in women even after adjusting for age, PEA, biological and behavioral risk factors and SES (in fully adjusted model 6: ß (SE) = −0.13(0.05), *p* = 0.003). *ADIPOQ* SNP rs1501299 was only significant in the crude model and the one adjusted for age. There were no association with the *ADIPOQ* SNPs and blood glucose in men or women as indicated in Table 5. Two variants were observed to be significantly associated with HOMA-IR in men. *ADIPOQ* SNP rs12495941 was significantly associated after adjusting for age, PEA, biological risk factors and behavioral risk factors, but the association attenuated and became marginally non-significant after adjusting for SES (model 6: ß (SE) = 0.40 (0.15), *p* =0.0086). However, the association between *ADIPOQ* SNP rs7627128 remained significant even when fully adjusted for SES (model 6: ß (SE) = −0.73 (0.20), *p* = 0.0003). Table 6 shows one variant was associated with HOMA-IR in women. *ADIPOQ* SNP rs1501299 was only significant in the crude and age adjusted models (*p* = 0.003 and 0.003, respectively). Table 7 reveals that there was no association between any of the variants and BMI in men or women.

**Table 3:** Association between Type 2 diabetes and *ADIPOQ* SNPs in men and women in the Jackson Heart Study, *N* = 2,978*

Men, *n* = 1,133													
SNPs	Alleles	Model 1^┼^		Model 2^╪^		Model 3^§^		Model 4^║^		Model 5^¶^		Model 6^#^	
		OR (95 % CI)	*P*-value	OR (95 % CI)	*P*-value	OR (95 % CI)	*P*-value	OR (95 % CI)	*P*-value	OR (95 % CI)	*P*-value	OR (95 % CI)	*P*-value
rs16861205	G/A	1.37 (0.89,2.10)	0.1532	1.44 (0.92,2.23)	0.1075	1.93 (1.01,3.53)	0.0322	1.52 (0.78,2.96)	0.2154	1.49 (0.75,2.96)	0.2517	1.51 (0.75,3.06)	0.2502
rs12495941	G/T	0.47 (0.19,1.14)	0.0932	0.52 (0.21,1.29)	0.1560	0.33 (0.11,0.99)	0.0489	0.54 (0.09,3.45)	0.5182	0.54 (0.08,3.51)	0.5149	0.42 (0.06,2.97)	0.3833
rs7627128	C/A	0.82 (0.19,3.52)	0.7895	1.06 (0.24,4.75)	0.9434	3.18 (0.18,56.0)	0.4301	8.35 (0.18,442)	0.2696	11.01 (0.19,621)	0.2413	12.55 (0.21,737)	0.2234
rs9877202	A/G	0.97 (0.61,1.54)	0.8969	0.96 (0.60,1.54)	0.8642	0.95 (0.54,1.68)	0.8648	0.98 (0.50,1.90)	0.9477	0.80 (0.40,1.60)	0.5230	0.82 (0.41,1.66)	0.5828
rs2036373	T/G	0.20 (0.01,5.07)	0.3303	0.13 (0.01,3.63)	0.2304	0.03 (<0.001,1.4)	0.0727	0.025 (<0.001, 1.12)	0.0571	0.015 (<0.001,0.79)	0.0376	0.018 (<0.001,0.964	0.0479
rs1501299	G/T	1.09 (0.66,1.82)	0.7310	1.10 (0.66,1.86)	0.7111	0.93 (0.49,1.76)	0.8240	0.73 (0.36,1.45)	0.3658	0.68 (0.34,1.39)	0.2913	0.71 (0.35,1.45)	0.3413
rs3821799	T/C	1.06 (0.77,1.44)	0.7308	1.02 (0.75,1.41)	0.8814	1.01 (0.72,1.62)	0.7151	1.00 (0.62,1.61)	0.9976	1.02 (0.62,1.69)	0.9313	0.96 (0.57,1.61)	0.8762
rs9842733	A/T	3.96 (0.43,36)	0.2224	4.40 (0.44,43.89)	0.2073	26.62 (0.174,>999)	0.2009	775 (0.009,>999)	0.2526	739 (0.008,>999)	0.2571	344 (0.008,>999)	0.2835
Women, *n* = 1,845													
SNPs	Alleles	Model 1┼		Model 2^╪^		Model 3^§^		Model 4^║^		Model 5^¶^		Model 6^#^	
		OR (95 % CI)	*P*-value	OR (95 % CI)	*P*-value	OR (95 % CI)	*P*-value	OR (95 % CI)	*P*-value	OR (95 % CI)	*P*-value	OR (95 % CI)	*P*-value
rs16861205	G/A	1.12 (0.80,1.57)	0.5021	1.15 (0.85,1.56)	0.3648	1.15 (0.78,1.68)	0.4872	1.21 (0.77,1.89)	0.4096	1.13 (0.70,1.81)	0.6175	1.17 (0.72,1.89)	0.5274
rs12495941	G/T	1.03 (0.54,1.97)	0.9361	1.15 (0.60,2.21)	0.6819	1.89 (0.58,6.17)	0.2909	2.85 (0.52,15.5)	0.2259	2.62 (0.47,14.5)	0.2703	3.15 (0.56,17.75)	0.1935
rs7627128	C/A	0.57 (0.20,1.66)	0.2993	0.73 (0.24,2.20)	0.5756	0.67 (0.14,3.17)	0.6127	1.58 (0.16,15.7)	0.6985	1.05 (0.10,11.1)	0.9705	1.07 (0.10,11.93)	0.9562
rs9877202	A/G	0.83 (0.61,1.15)	0.2601	0.81 (0.59,1.12)	0.1989	0.91 (0.59,1.40)	0.6607	0.94 (0.55,1.58)	0.8034	1.05 (0.58,1.88)	0.8847	1.04 (0.58,1.88)	0.8863
rs2036373	T/G	2.56 (0.09,75)	0.5870	1.56 (0.05,51)	0.8028	0.95 (0.03,28)	0.9781	2.48 (0.02,255)	0.7004	1.10 (0.01,140)	0.9685	1.17 (0.01,141)	0.9484
rs1501299	G/T	1.50 (0.97,2.30)	0.0718	1.41 (0.91,2.2)	0.1237	1.33 (0.78,2.28)	0.2949	1.44 (0.75,2.76)	0.2766	2.34 (1.08, 5.06)	0.0309	2.46 (1.13, 5.36)	0.0232
rs3821799	T/C	0.93 (0.75,1.17)	0.5489	0.93 (0.74,1.17)	0.5445	0.84 (0.63,1.12)	0.2347	0.85 (0.60,1.19)	0.3359	0.83 (0.57,1.19)	0.3074	0.82 (0.57,1.19)	0.3018
rs9842733	A/T	0.71 (0.30,1.67)	0.4362	0.85 (0.36,2.04)	0.7187	0.76 (0.26,2.17)	0.6019	0.78 (0.23,2.62)	0.6816	0.58 (0.15,2.26)	0.4308	0.59 (0.15,2.33)	0.4529

* N represents 42 missing values for type 2 diabetes

^┼^Model 1: crude

^╪^Model 2: adjusted for age

^§^Model 3: adjusted for age, PEA

^║^Model 4: adjusted for age, PEA, LDL, HDL, triglyceride, CRP, plasma leptin

^¶^Model 5: adjusted for age, PEA, LDL, HDL, triglyceride, CRP, plasma leptin, smoking status, physical activity score, BMI, alcohol consumption status

^#^Model 6: adjusted for age, PEA, LDL, HDL, triglyceride, CRP, plasma leptin,, smoking status, physical activity score, BMI, alcohol consumption status, socioeconomic status (education level)

Two-tailed level of significance was established as *P* ≤ 0.006

**Table 4:** Association between plasma adiponectin level and *ADIPOQ* SNPs among men and women in the Jackson Heart Study, *N* = 2,968*

Men, *n* = 1,131													
SNPs	Alleles	Model 1^┼^		Model 2^╪^		Model 3^§^		Model 4^║^		Model 5^¶^		Model 6^#^	
		β (SE)	*P*-value	β (SE)	*P*-value	β (SE)	*P*-value	β (SE)	*P*-value	β (SE)	*P*-value	β (SE)	*P*-value
rs16861205	G/A	−0.10(0.05)	0.0578	−0.09(0.05)	0.0652	−0.14(0.06)	0.0124	−0.09(0.05)	0.1075	−0.10(0.06)	0.0603	−0.10(0.06)	0.0914
rs12495941	G/T	−0.12(0.14)	0.3822	−0.08(0.14)	0.5438	−0.12(0.16)	0.4513	0.04(0.17)	0.8283	−0.01(0.18)	0.9377	−0.03(0.18)	0.8546
rs7627128	C/A	−0.19(0.20)	0.3345	−0.12(0.19)	0.5436	−0.13(0.26)	0.6145	−0.25(0.24)	0.2962	−0.30(0.24)	0.2086	−0.31(0.24)	0.1968
rs9877202	A/G	−0.08(0.06)	0.1709	−0.08(0.06)	0.1652	−0.10(0.06)	0.1192	−0.06(0.06)	0.3117	−0.09(0.06)	0.1764	−0.10(0.07)	0.1207
rs2036373	T/G	−1.12(0.52)	0.0335	−1.16(0.51)	0.0240	−0.92(0.57)	0.1080	−0.14(0.56)	0.7934	−0.47(0.60)	0.4295	−0.52(0.59)	0.3827
rs1501299	G/T	−0.01(0.06)	0.9324	−0.01(0.06)	0.8870	−0.06(0.07)	0.410	−0.12(0.06)	0.0719	−0.13(0.07)	0.0533	−0.14(0.07)	0.0491
rs3821799	T/C	0.04(0.04)	0.3710	0.03(0.04)	0.4916	0.02(0.04)	0.6712	0.007(0.04)	0.8744	−0.01(0.04)	0.9014	−0.01(0.04)	0.8580
rs9842733	A/T	−0.17(0.20)	0.3862	−0.19(0.19)	0.3223	−0.33(0.22)	0.1436	−0.20(0.20)	0.3165	−0.32(0.24)	0.1378	−0.33(0.21)	0.1284
Women, *n* = 1,837													
SNPs	Alleles	Model 1^┼^		Model 2^╪^		Model 3^§^		Model 4^║^		Model 5^¶^		Model 6^#^	
		β (SE)	*P*-value	β (SE)	*P*-value	β (SE)	*P*-value	β (SE)	*P*-value	β (SE)	*P*-value	β (SE)	*P*-value
rs16861205	G/A	−0.14(0.04)	0.0001	−0.14(0.04)	0.0002	−0.11(0.04)	0.0089	−0.10(0.04)	0.017	−0.13(0.05)	0.006	−0.13(0.05)	0.003
rs12495941	G/T	0.09(0.08)	0.3047	0.12(0.08)	0.1551	0.008(0.12)	0.9416	0.06(0.10)	0.5292	0.07(0.11)	0.5227	0.07(0.11)	0.5428
rs7627128	C/A	−0.06(0.15)	0.6956	−0.01(0.15)	0.9514	−0.15(0.20)	0.4585	−0.32(0.19)	0.0874	−0.58(0.23)	0.0117	−0.61(0.23)	0.0084
rs9877202	A/G	−0.05(0.04)	0.2295	−0.05(0.04)	0.1939	−0.05(0.05)	0.3580	−0.03(0.05)	0.5442	−0.07(0.05)	0.1939	−0.07(0.06)	0.1789
rs2036373	T/G	0.21(0.37)	0.5682	0.10(0.36)	0.7810	0.23(0.37)	0.5343	0.25(0.33)	0.4459	0.43(0.39)	0.2669	0.42(0.39)	0.2745
rs1501299	G/T	−0.14(0.05)	0.004	−0.15(0.05)	0.001	−0.13(0.06)	0.0258	−0.03(0.05)	0.510	−0.12(0.06)	0.0472	−0.12(0.06)	0.0468
rs3821799	T/C	0.03(0.03)	0.2802	0.03(0.03)	0.2768	0.02(0.03)	0.4649	0.02(0.03)	0.5966	0.02(0.04)	0.5226	0.03(0.04)	0.4464
rs9842733	A/T	−0.15(0.12)	0.2107	−0.10(0.12)	0.3741	0.05(0.14)	0.6928	0.02(0.14)	0.9038	−0.01(0.16)	0.9561	0.005(0.16)	0.9748

^*^N represents 52 missing values for adiponectin

^┼^Model 1: crude

^╪^Model 2: adjusted for age

^§^Model 3: adjusted for age, PEA

^║^Model 4: adjusted for age, PEA, LDL, HDL, triglyceride, CRP, plasma leptin, blood glucose, HOMA-IR

^¶^Model 5: adjusted for age, PEA, LDL, HDL, triglyceride, CRP, plasma leptin, blood glucose, HOMA-IR, smoking status, physical activity score, BMI, alcohol consumption status

^#^Model 6: adjusted for age, PEA, LDL, HDL, triglyceride, CRP, plasma leptin, blood glucose, HOMA-IR, smoking status, physical activity score, BMI, alcohol consumption status, socioeconomic status (education level)

Two-tailed level of significance established as *P* ≤ 0.006

**Table 5:** Association between blood glucose and *ADIPOQ* SNPs among men and women in the Jackson Heart Study, *N* = 2,800*

Men, *n* = 1,071													
SNPs	Alleles	Model 1^┼^		Model 2^╪^		Model 3^§^		Model 4^║^		Model 5^¶^		Model 6^#^	
		β (SE)	*P*-value	β (SE)	*P*-value	β (SE)	*P*-value	β (SE)	*P*-value	β (SE)	*P*-value	β (SE)	*P*-value
rs16861205	G/A	0.01(0.02)	0.4877	0.02(0.02)	0.3755	0.03(0.02)	0.2168	0.01(0.02)	0.6174	0.004(0.02)	0.8347	0.005(0.02)	0.8140
rs12495941	G/T	0.03(0.05)	0.5088	0.04(0.04)	0.3695	0.03(0.06)	0.6776	−0.001(0.06)	0.9887	−0.02(0.07)	0.7920	−0.03(0.07)	0.6549
rs7627128	C/A	−0.05(0.07)	0.4997	−0.03(0.001)	0.7078	−0.01(0.09)	0.9074	0.02(0.09)	0.8449	0.03(0.09)	0.7506	0.04(0.09)	0.6765
rs9877202	A/G	−0.01(0.02)	0.7960	−0.01(0.02)	0.7940	−0.01(0.02)	0.6964	−0.004(0.02)	0.8658	−0.01(0.02)	0.6983	−0.01(0.02)	0.5648
rs2036373	T/G	−0.19(0.19)	0.3200	−0.20(0.18)	0.2764	−0.38(0.21)	0.0694	−0.38(0.20)	0.0489	−0.46(0.21)	0.0262	−0.45(0.20)	0.0289
rs1501299	G/T	−0.01(0.02)	0.6674	−0.01(0.02)	0.6454	−0.001(0.03)	0.9726	−0.01(0.02)	0.6306	−0.01(0.03)	0.6235	−0.01(0.03)	0.6798
rs3821799	T/C	0.005(0.01)	0.7486	0.002(0.01)	0.8970	0.0004(0.02)	0.9764	0.01(0.02)	0.6961	0.01(0.02)	0.6086	0.01(0.02)	0.6701
rs9842733	A/T	0.09(0.07)	0.2152	0.08(0.07)	0.2207	0.06(0.08)	0.4388	0.04(0.08)	0.6100	0.05(0.08)	0.5668	0.05(0.08)	0.5539
Women, *n* = 1,729													
SNPs	Alleles	Model 1^┼^		Model 2^╪^		Model 3^§^		Model 4^║^		Model 5^¶^		Model 6^#^	
		β (SE)	*P*-value	β (SE)	*P*-value	β (SE)	*P*-value	β (SE)	*P*-value	β (SE)	*P*-value	β (SE)	*P*-value
rs16861205	G/A	0.02(0.02)	0.1881	0.004(0.01)	0.1790	0.01(0.02)	0.3971	0.02(0.02)	0.1640	0.02(0.02)	0.2073	0.02(0.02)	0.1810
rs12495941	G/T	−0.003(0.03)	0.9296	0.01(0.03)	0.7260	0.04(0.04)	0.4129	0.02(0.04)	0.6438	0.01(0.04)	0.7305	0.01(0.04)	0.7427
rs7627128	C/A	−0.01(0.06)	0.8482	0.02(0.06)	0.7974	0.08(0.08)	0.2949	0.11(0.08)	0.1639	0.10(0.08)	0.2094	0.09(0.08)	0.2248
rs9877202	A/G	−0.03(0.02)	0.0672	−0.04(0.02)	0.0338	−0.01(0.02)	0.4711	−0.02(0.02)	0.2251	−0.02(0.02)	0.3394	−0.02(0.02)	0.3012
rs2036373	T/G	0.07(0.14)	0.6170	0.03(0.14)	0.8356	−0.01(0.14)	0.9198	0.01(0.13)	0.9309	−0.03(0.16)	0.8586	−0.03(0.16)	0.8422
rs1501299	G/T	0.02(0.02)	0.3343	0.02(0.02)	0.4112	0.002(0.02)	0.9379	−0.01(0.02)	0.5848	−0.01(0.02)	0.7571	−0.01(0.02)	0.7473
rs3821799	T/C	0.01(0.01)	0.4056	0.01(0.01)	0.4642	−0.003(0.01)	0.8430	0.01(0.01)	0.6920	0.01(0.01)	0.6938	0.01(0.01)	0.6240
rs9842733	A/T	0.02(0.05)	0.6652	0.03(0.05)	0.4631	0.02(0.05)	0.7694	0.04(0.05)	0.4431	0.02(0.05)	0.6909	0.03(0.05)	0.6373

^*^N represents 220 missing values for blood glucose

^┼^Model 1: crude

^╪^Model 2: adjusted for age

^§^Model 3: adjusted for age, PEA

^║^Model 4: adjusted for age, PEA, LDL, HDL, triglyceride, CRP, plasma leptin

^¶^Model 5: adjusted for age, PEA, LDL, HDL, triglyceride, CRP, plasma leptin, smoking status, physical activity score, BMI, alcohol consumption status

^#^Model 6: adjusted for age, PEA, LDL, HDL, triglyceride, CRP, plasma leptin,, smoking status, physical activity score, BMI, alcohol consumption status, socioeconomic status (education level)

Two-tailed level of significance was established as *P* ≤ 0.006

**Table 6:** Association between HOMA-IR and *ADIPOQ* SNPs among men and women in the Jackson Heart Study, *N* = 2,347*

Men, *n* = 920													
SNPs	Alleles	Model 1^┼^		Model 2^╪^		Model 3^§^		Model 4^║^		Model 5^¶^		Model 6^#^	
		β (SE)	*P*-value	β (SE)	*P*-value	β (SE)	*P*-value	β (SE)	*P*-value	β (SE)	*P*-value	β (SE)	*P*-value
rs16861205	G/A	0.04(0.05)	0.3782	0.04(0.05)	0.3939	0.04(0.06)	0.4916	−0.03(0.05)	0.5570	−0.04(0.05)	0.4126	−0.05(0.05)	0.3641
rs12495941	G/T	0.43(0.14)	0.001	0.43(0.14)	0.002	0.76(0.18)	<.0001	0.41(0.15)	0.004	0.42(0.15)	0.005	0.40(0.15)	0.0086
rs7627128	C/A	−0.98(0.20)	<.0001	−0.99(0.20)	<.0001	−0.13(0.26)	<.0001	−0.78(0.20)	0.0001	−0.74(0.20)	0.0002	−0.73(0.20)	0.0003
rs9877202	A/G	−0.002(0.06)	0.9784	−0.001(0.06)	0.9913	−0.02(0.07)	0.7451	−0.03(0.05)	0.5270	−0.04(0.06)	0.4308	−0.05(0.06)	0.3797
rs2036373	T/G	0.42(0.51)	0.4158	0.42(0.51)	0.4102	0.06(0.63)	0.9262	−0.15(0.49)	0.7626	−0.05(0.52)	0.9197	−0.07(0.52)	0.8889
rs1501299	G/T	−0.05(0.06)	0.3976	−0.05(0.06)	0.3886	−0.03(0.07)	0.7110	−0.02(0.06)	0.7035	−0.04(0.06)	0.5522	−0.03(0.06)	0.6309
rs3821799	T/C	0.03(0.04)	0.4645	0.03(0.04)	0.4422	0.05(0.05)	0.2534	0.08(0.04)	0.0258	0.07(0.04)	0.0638	0.07(0.04)	0.0608
rs9842733	A/T	0.11(0.18)	0.5344	0.12(0.18)	0.5306	0.17(0.26)	0.4427	0.18(0.18)	0.3151	0.07(0.19)	0.6947	0.07(0.19)	0.6955
Women, *n* = 1,427													
SNPs	Alleles	Model 1^┼^		Model 2^╪^		Model 3^§^		Model 4^║^		Model 5^¶^		Model 6^#^	
		β(SE)	*P*-value	β(SE)	*P*-value	β(SE)	*P*-value	β(SE)	*P*-value	β(SE)	*P*-value	β(SE)	*P*-value
rs16861205	G/A	0.07(0.04)	0.0795	0.07(0.04)	0.0776	0.06(0.04)	0.1816	0.07(0.04)	0.0491	0.07(0.04)	0.0594	0.07(0.04)	0.0624
rs12495941	G/T	0.11(0.08)	0.1814	0.12(0.08)	0.1510	0.11(0.11)	0.3332	0.01(0.10)	0.8898	0.01(0.10)	0.9282	0.004(0.10)	0.9626
rs7627128	C/A	−0.12(0.16)	0.4349	−0.11(0.16)	0.4908	0.001(0.21)	0.9959	0.08(0.17)	0.6421	0.05(0.17)	0.7616	0.05(0.17)	0.7874
rs9877202	A/G	−0.01(0.05)	0.8391	−0.01(0.05)	0.7704	0.05(0.05)	0.3250	0.01(0.04)	0.8426	0.01(0.05)	0.7866	0.01(0.05)	0.8183
rs2036373	T/G	−0.01(0.34)	0.9876	−0.02(0.34)	0.9438	−0.05(0.36)	0.8922	−0.002(0.3)	0.9933	−0.11(0.36)	0.7577	−0.10(0.36)	0.7696
rs1501299	G/T	0.15(0.05)	0.003	0.14(0.05)	0.003	0.13(0.06)	0.0226	0.06(0.05)	0.2300	0.08(0.05)	0.0996	0.08(0.05)	0.1006
rs3821799	T/C	0.01(0.03)	0.8004	0.01(0.03)	0.7921	−0.01(0.04)	0.7620	−0.02(0.03)	0.5097	−0.03(0.03)	0.3304	−0.03(0.03)	0.3290
rs9842733	A/T	0.30(0.16)	0.0176	0.31(0.13)	0.0139	0.29(0.15)	0.0539	0.33(0.13)	0.0098	0.27(0.13)	0.0385	0.28(0.13)	0.0337

^*^N represents 673 missing values for HOMA-IR

^┼^Model 1: crude

^╪^Model 2: adjusted for age

^§^Model 3: adjusted for age, PEA

^║^Model 4: adjusted for age, PEA, LDL, HDL, triglyceride, CRP, plasma leptin

^¶^Model 5: adjusted for age, PEA, LDL, HDL, triglyceride, CRP, plasma leptin, smoking status, physical activity score, BMI, alcohol consumption status

^#^Model 6: adjusted for age, PEA, LDL, HDL, triglyceride, CRP, plasma leptin, smoking status, physical activity score, BMI, alcohol consumption status, socioeconomic status (education level)

Two-tailed level of significance established as *P* ≤ 0.006

**Table 7:** Association between BMI and *ADIPOQ* SNPs among men and women in the Jackson Heart Study, *N* = 3,015*

Men, n = 1,150													
SNPs	Alleles	Model 1^┼^		Model 2^╪^		Model 3^§^		Model 4^║^		Model 5^¶^		Model 6^#^	
		β (SE)	*P*-value	β (SE)	*P*-value	β (SE)	*P*-value	β(SE)	*P*-value	β (SE)	*P*-value	β (SE)	*P*-value
rs16861205	G/A	0.02(0.01)	0.2907	0.01(0.01)	0.3250	0.02(0.02)	0.2014	0.001(0.01)	0.9212	0.001(0.01)	0.9286	−0.01(0.01)	0.9369
rs12495941	G/T	0.04(0.04)	0.3020	0.03(0.04)	0.3923	0.06(0.05)	0.2288	−0.02(0.04)	0.5470	−0.04(0.04)	0.3368	−0.04(0.04)	0.2705
rs7627128	C/A	−0.09(0.06)	0.1112	−0.10(0.06)	0.0656	−0.12(0.08)	0.1383	−0.01(0.05)	0.9122	−0.01(0.05)	0.9111	−0.002(0.05)	0.9631
rs9877202	A/G	0.01(0.02)	0.4062	0.01(0.02)	0.3984	0.002(0.02)	0.8888	−0.003(0.01)	0.8100	0.004(0.01)	0.7816	0.006(0.01)	0.7000
rs2036373	T/G	0.18(0.15)	0.2362	0.19(0.15)	0.2090	0.09(0.18)	0.6042	0.03(0.12)	0.8097	0.06(0.13)	0.6559	0.06(0.13)	0.6333
rs1501299	G/T	−0.01(0.02)	0.5381	−0.01(0.02)	0.5427	−0.02(0.02)	0.4453	−0.003(0.02)	0.8198	−0.01(0.02)	0.7433	−0.004(0.02)	0.7959
rs3821799	T/C	0.01(0.01)	0.5231	0.01(0.01)	0.4373	0.01(0.01)	0.4451	0.01(0.01)	0.3572	0.01(0.01)	0.2271	0.01(0.01)	0.3012
rs9842733	A/T	−0.05(0.06)	0.4189	−0.04(0.06)	0.4565	−0.03(0.07)	0.6265	0.01(0.05)	0.8381	0.02(0.05)	0.6911	0.01(0.05)	0.7695
Women, *n* = 1,865													
SNPs	Alleles	Model 1^┼^		Model 2^╪^		Model 3^§^		Model 4^║^		Model 5^¶^		Model 6^#^	
		β (SE)	*P*-value	β (SE)	*P*-value	β (SE)	*P*-value	β (SE)	*P*-value	β (SE)	*P*-value	β (SE)	*P*-value
rs16861205	G/A	0.01(0.01)	0.5560	0.01(0.01)	0.5908	0.02(0.02)	0.3135	0.01(0.01)	0.5001	0.002(0.01)	0.8239	0.004(0.01)	0.7495
rs12495941	G/T	0.06(0.03)	0.0447	0.05(0.03)	0.0724	0.05(0.04)	0.2977	0.01(0.03)	0.7523	0.01(0.03)	0.6878	0.01(0.03)	0.7813
rs7627128	C/A	0.001(0.05)	0.9921	−0.01(0.05)	0.8213	0.06(0.07)	0.4078	0.09(0.06)	0.1436	0.09(0.06)	0.1172	0.09(0.06)	0.1307
rs9877202	A/G	−0.01(0.02)	0.7077	−0.01(0.02)	0.7377	0.002(0.02)	0.9166	−0.01(0.01)	0.3950	−0.01(0.02)	0.5705	−0.01(0.02)	0.5568
rs2036373	T/G	−0.07(0.13)	0.5655	−0.05(0.13)	0.6915	−0.05(0.14)	0.6920	−0.05(0.11)	0.6477	−0.02(0.12)	0.8414	−0.03(0.12)	0.7874
rs1501299	G/T	0.01(0.02)	0.7208	0.01(0.02)	0.6198	0.01(0.02)	0.5665	−0.01(0.02)	0.4194	−0.02(0.02)	0.3642	−0.02(0.02)	0.3543
rs3821799	T/C	0.002(0.01)	0.8623	0.002(0.01)	0.8598	0.005(0.01)	0.7169	−0.0001(0.01)	0.9897	0.003(0.01)	0.7858	0.004(0.01)	0.7302
rs9842733	A/T	0.03(0.04)	0.4526	0.02(0.04)	0.5909	0.07(0.05)	0.1991	0.06(0.04)	0.1338	0.06(0.04)	0.1485	0.06(0.04)	0.1391

^*^N represents 5 missing values for BMI

^┼^Model 1: crude

^╪^Model 2: adjusted for age

^§^Model 3: adjusted for age, PEA

^║^Model 4: adjusted for age, PEA, LDL, HDL, triglyceride, CRP, plasma peptin

^¶^Model 5: adjusted for age, PEA, LDL, HDL, triglyceride, CRP, plasma leptin, smoking status, physical activity score, alcohol consumption status

^#^Model 6: adjusted for age, PEA, LDL, HDL, triglyceride, CRP, plasma leptin, smoking status, physical activity score, alcohol consumption status, socioeconomic status (education level)

Two-tailed level of significance established as *P* ≤ 0.006

### Association between haplotypes with HOMA-IR and adiponectin

SNPs that were significantly associated with HOMA-IR and adiponectin (rs7627128 and rs16861205) were tested. The haplotype analysis did not reveal any significant association after controlling for covariates (data not shown).

## Discussion

Selected *ADIPOQ* SNPs were analyzed to assess their association with type 2 diabetes and related phenotypes in a large well characterized sample of African Americans. Our findings show the *ADIPOQ* variant rs16861205 (MAF = 0.21) was significantly associated with a lower level of plasma adiponectin in women with minor allele A than none-carriers. This association was attenuated after adjusting for PEA and biological risk factors but persisted when fully adjusted for age, PEA, biological and behavioral risk factors and SES. These findings suggest an etiological association between genetic variant rs16861205 and lower levels of adiponectin observed in African American women either directly or through another variant that is linked to it. Gender can be considered a measured environmental risk factor which incorporates established anatomical, physiological, and behavioral differences between genders. The gender dismorphism in adiponectin levels is well established starting at puberty - possibly influenced by sex hormones which might explain our observation of lower adiponectin in women [32]. Our findings of observed lower levels of adiponectin in women are consistent with other research that similarly document lower levels of adiponectin in African American women when compared to other race/ethnic women [32, 36]. Cohen et al., for instance, observed a lower level of serum adiponectin in African American women when compared to white women [36]. However, unlike our finding, they did not find any associations between adiponectin and the SNPs in the adiponectin gene that were assessed. This observation may be due to a smaller sample size. *ADIPOQ* variant rs1501299 in women with minor allele T also had lower plasma adiponectin after adjusting for age, but this association disappeared after adjusting for PEA, biological and behavioral risk factors and SES.

Our findings also revealed that the *ADIPOQ* SNP rs12495941 (MAF = 0.35) was significantly associated with higher HOMA-IR among men with carriers of the minor allele T suggesting perhaps a relationship between the variant and likelihood of type 2 diabetes. The rs1249541variant is located in the intron 1 region not involved in any putative transcription factor binding site which means this SNP is a noncoding variant without obvious regulatory function. Thus, this SNP may be in linkage disequilibrium with another functional variant in African Americans [15]. We attempted to predict in sylico the potential functionality of the tagged SNPS with software AliBaba in order to test their role as potential transcriptional regulators of adiponectin expression through different mechanisms such as sequence alterations involved splicing processes and modifications in transcriptional factors binding motifs [37]. Our analysis revealed the tested SNPs disrupted or resulted in the appearance of putative transcription factor binding sites. Further functional analysis studies of this and other SNPs, particularly in African Americans, are needed to elucidate the potential role in regulating adiponectin expression.

*ADIPOQ* SNP rs1249541 has been found to be associated with adiponectin levels and anthropomorphic measures in other populations [38]. However, to the best of our knowledge, this is the first report on a gender specific association between the rs12495941 variant and HOMA-IR. The Bonferroni significance threshold, however, was lost in the model that was fully adjusted for SES. On the other hand, the *ADIPOQ* SNP rs7627128 was also associated with HOMA-IR in male carriers of the minor allele who had significantly lower HOMA-IR, and this finding was consistent in each of the models. As with rs1249541, this SNP is located in the intron 1 region and lacks obvious regulatory function and also represents a novel finding. *ADIPOQ* SNP rs1501299 in women with minor allele T had higher HOMA-IR but this association did not persist beyond adjustment for age. This attenuation underscores the importance of including adjustment for African ancestry (model 3) in analyses of African American populations. The association at different SNPs in our sample is not unexpected. Ukkola et al. indicate this may be a reflection of ethnic differences in adiponectin gene structure based on their evaluation of African Americans from the HERITAGE study [38]. The data in their study are further supported by evidence demonstrating African Americans have reduced plasma adiponectin concentrations when compared to other ethnic groups [10]. The potential for ethnic differences in the adiponectin gene emphasizes the need to study genetic associations in a variety of populations. The differential sex observation related to SNPs rs12495941 and rs7627128 is not clearly understood, but may be related to sex-specific hormones such as estradiol and testosterone as observed with rs16861205 and adiponectin [39–41]. There was substantial missing HOMA-IR data in our data which may likewise result in biased findings. Further research on *ADIPOQ* variants and HOMA-IR on both sexes accounting for sex hormones is warranted to elucidate the biological mechanisms of this association.

Our study did not reveal any association of *ADIPOQ* SNPs with type 2 diabetes, blood glucose or BMI in men or women. These findings are interesting given prior evidence documenting the association of *ADIPOQ* gene with type 2 diabetes, insulin resistance, elevated blood glucose and BMI [12–15]. However, such reports did not adequately control for ancestry, biological and behavioral risk factors or SES when assessing the association of *ADIPOQ* polymorphisms. An investigation by Bostrom et al., for instance, similarly found that SNP rs3821799 in the *ADIPOQ* gene was not associated with type 2 diabetes in African Americans [15]. These investigators also tested the association of SNP rs1501299 and found no association with type 2 diabetes. Previous studies that did not include African Americans detected an association of *ADIPOQ* SNPs in the promoter region or in exons (exon 3) with morbid obesity and with type 2 diabetes [42, 43]. Our analysis of variant rs12495941 revealed no associations with our outcomes. A study of this variant in a sample of Indians also found no association with type 2 diabetes or insulin sensitivity related variables [44]. This polymorphism was, however, associated with fasting glucose levels in Hispanics [45]. We also assessed variant rs9877202. Few studies have investigated this intronic polymorphism. However, this variant was not associated with any study outcomes [46]. A recent meta-analysis reported a genetic susceptibility for type 2 diabetes linked to rs1501299 in East Asian populations [47]. We found no association with any of the outcomes in our study.

Several studies have reported a significant association between BMI and various *ADIPOQ* SNPs [17, 36, 38], albeit with inconsistent results across studies. Furthermore, such studies were conducted in non-African American populations and did not report sex differences. However, evidence from a genome wide association study by Liu et al. identified two waist-related genetic loci (LHX2 and RREB1) associated with fat distribution in African American populations [17]. A report by An et al. of the IRAS Family Study, on the other hand, indicates no association between selected *ADIPOQ* SNPs and BMI in African Americans [42]. They further report that only one promoter SNP was positively associated with plasma adiponectin and fasting glucose in African Americans – rs17300539.

### Strengths and limitations

The main strength of this investigation is that findings were from the largest community-based sample of African Americans, a cohort with strict protocol and high quality-control. It also addresses a health outcome that disproportionately affects African Americans. In addition, it presents differential findings between African American men and women. Further, the sample size far exceeds those in previous reports and the study used a tag SNP approach that captures much of the variation across the adiponectin gene in African Americans. The analysis was also adjusted for global/aggregate genetic ancestry, biological and behavioral risk factors and socioeconomic status. In terms of limitations, findings cannot be generalized to other ethnic groups. Secondly, this is a cross-sectional analysis and causality between *ADIPOQ* SNPs and phenotypes cannot be attributed without longitudinal tracking or incidence. Finally, although some of the associated SNPs did not reach a Bonferroni-adjusted threshold of significance, it will be important to replicate these findings in additional suitable cohorts.

## Conclusion

The objective of this study was to assess the association of tag *ADIPOQ* SNPs with type 2 diabetes and related phenotypes between African American men and women. No association was observed between *ADIPOQ* SNPs and type 2 diabetes, blood glucose or BMI in men or women. A significant association with variant rs16861205 and lower adiponectin level was revealed in women with minor allele A. Variant rs12495941 revealed men with minor allele T had higher HOMA-IR but significance disappeared after adjustment for SES. Variant rs7627128 indicated men with minor allele A had significantly lower HOMA-IR that remained consistent in the fully adjusted model. These associations represent novel findings. As with any gene-phenotype association study, it is necessary to replicate study findings in other large well characterized study populations. Our well-adjusted findings nevertheless suggest important new insights regarding the association between *ADIPOQ* SNPs and type 2 diabetes and related phenotypes in African American men and women a disproportionately affected population.

## Availability of supporting data

Data for this study were deposited in the National Institutes of Health The database of Genotypes and Phenotypes (DbGAP) found at www.ncbi.nlm.gov/gap/?item=Jackson+Heart+Study [48].
